# Animal-vehicle collisions during the COVID-19 lockdown in early 2020 in the Krakow metropolitan region, Poland

**DOI:** 10.1038/s41598-022-11526-9

**Published:** 2022-05-09

**Authors:** Sayantani M. Basak, Declan T. O’Mahony, Maciej Lesiak, Arpan Kumar Basak, Elżbieta Ziółkowska, Dominik Kaim, Md Sarwar Hossain, Izabela A. Wierzbowska

**Affiliations:** 1 Institute of Environmental Sciences, Faculty of Biology, Jagiellonian University, Gronostajowa 7, 30-387 Kraków, Poland; 2 Agri-Food and Biosciences Institute, 18a Newforge Lane, Belfast, BT9 6AT Northern Ireland; 3 “KABAN” Maciej Lesiak, Stare Wislisko 48, 31-979 Kraków, Poland; 4 Małopolskie Centrum of Biotechnology, Jagiellonian University, Gronostajowa 7A, 30-387 Kraków, Poland; 5 Institute of Geography and Spatial Management, Faculty of Geography and Geology, Jagiellonian University, Gronostajowa 7, 30-387 Kraków, Poland; 6 Environmental Science and Sustainability, School of Interdisciplinary Studies, University of Glasgow, Dumfries, DG1 4ZL UK

**Keywords:** Ecology, Ecology, Environmental sciences

## Abstract

The interrelations between human activity and animal populations are of increasing interest due to the emergence of the novel COVID-19 and the consequent pandemic across the world. Anthropogenic impacts of the pandemic on animals in urban-suburban environments are largely unknown. In this study, the temporal and spatial patterns of urban animal response to the COVID-19 lockdown were assessed using animal-vehicle collisions (AVC) data. We collected AVC data over two 6-month periods in 2019 and 2020 (January to June) from the largest metropolis in southern Poland, which included lockdown months. Furthermore, we used traffic data to understand the impact of lockdown on AVC in the urban area. Our analysis of 1063 AVC incidents revealed that COVID-19 related lockdown decreased AVC rates in suburban areas. However, in the urban area, even though traffic volume had significantly reduced, AVC did not decrease significantly, suggesting that lockdown did not influence the collision rates in the urban area. Our results suggest that there is a need to focus on understanding the effects of changes in traffic volume on both human behaviour and wildlife space use on the resulting impacts on AVC in the urban area.

## Introduction

Humans and wildlife have coexisted in urban environments for as long as human settlements have occurred^1^. Ever-expanding human populations have transformed environments at unprecedented rates, therefore, understanding the linkages between human and animal interactions are of critical importance given that human activities affect animal distribution, behaviour and movement^2,3^. Declared a global pandemic in March 2020, the outbreak of COVID-19 caused by the novel coronavirus SARS-CoV-2 in early 2020 brought about major changes to human dynamics on a global scale^4^. Governments restricted people’s mobility through stay at home requirements termed “lockdowns”^5^ to prevent the spread of disease^6^.

The COVID-19 pandemic and subsequent reductions in human activity have had considerable localised consequences for animals. For example, stress levels have been reduced on sensitive animal species in protected areas^7^ and there are reports of other species venturing into urban settings including pumas (*Felis concolor*)^8^, and golden jackals (*Canis aureus*)^9^. Urban birds responded to reduced noise pollution by producing songs at low amplitudes^10^ or increased their presence in the early morning hours^11^. On the other hand, however, increasing levels of illegal hunting due to reduced enforcement and protection measures have also occurred^12,13^. Globally, researchers have advocated for further research to understand the positive and negative effects resulting from COVID-related-human lockdown and its consequent relaxation (i.e., the Global Human Confinement Experiment^14^).

Scientists have long sought to quantify how human activities affect various aspects of wildlife ecology including movements and animal activity^9^. Studying the movement of animals in urban landscapes during a lockdown provides a unique opportunity to investigate one of the main drivers of this process^15,16^. Animal-vehicle collisions (AVC) are an important data source on animal movement and activity and are also pertinent to species conservation, human-wildlife conflict and human safety^17,18^. There have been a growing number of AVC cases worldwide due to expanded road networks and growing traffic volumes^19^, which are more intensified in urban environments^20,21^. Annually, an estimated 194 million birds and 29 million mammals are killed annually on European roads alone^22^ with ungulates alone exceeding 1 million per year^23^. In a recent study, Bíl^22^ found that wildlife vehicle collisions (WVC) had mostly reduced in many countries during the COVID-19 lockdown, albeit with varying rates. This broad study across Europe found that each country responded differently to reduced human mobility due to nationwide lockdowns.

The first case of COVID-19 in Poland was registered on 4 March 2020 and was declared an epidemic threat on 14 March 2020^24^. The government closed all educational facilities, including child care institutions along with prohibition of all public events ^24^. Gatherings of more than 2 people were banned, social distancing (2 m, subsequently reduced to 1.5 m) was required, mobility limited to a minimum, and the maximum capacity for public transport was reduced to half the number of seats^25^. Thus, the aim of this study was to identify the changes in AVC due to lockdown in an urban-suburban landscape during the COVID-19 pandemic in Poland. We collected AVC data over two six-month periods collected in the Krakow metropolitan area of Poland (which included urban commune of Krakow, and suburban communes of Wieliczka and Niepolomice). In our analyses, the months from January to June 2019 and, January, February and June 2020 were termed as ‘non-lockdown’ months. The lockdown period in 2020 was defined from March to May, hereafter termed ‘lockdown’ months based on the official government declaration^26^. Further, in our analyses, we used traffic data of Krakow to understand the impact of lockdown on AVC in the urban area. Both 2019 and 2020 were similar in terms of meteorological conditions in winter (January–March) and spring months (April–June) and there were also no essential landcover or human populations changes between these periods, controlling for any potential effects of such variables on analytical outcomes. Our two main research questions of this study were to investigate whether (1) lockdown influenced the incidence of AVC between the urban and suburban areas, and (2) if AVC within the urban commune of Krakow decreased with the traffic volume during the lockdown.

## Results

A total of 1063 AVC incidents involving avian (hereafter birds) and 15 wildlife mammalian species were recorded during the study period (see Table 1). As it was not possible to distinguish between smaller bird species, we grouped birds into a taxonomic group, which comprised 170 AVC cases. As for mammals, roe deer (*Capreolus capreolus*) (*n* = 519), red fox (*Vulpes vulpes*) (*n* = 117), wild boar (*Sus scrofa*) (*n* = 88), northern white-breasted hedgehog (hereafter hedgehog; *Erinaceus roumanicus*) (*n* = 62), brown hare (*Lepus europaeus*) (*n* = 35) and red squirrel (*Sciurus vulgaris*) (*n* = 21) were most frequently involved in road collisions in Krakow, Wieliczka and Niepolomice

**Table 1:** The total and mean (± SD) animal-vehicle collision (AVC) number during non-lockdown (January, February and June) and lockdown periods (March, April and May) recorded in the study area.

Commune	Year	Period	Total AVC	Mean AVC (per month)	SD
Krakow	2019	Non lockdown months	267	89.00	38.94
		Lockdown months	390	130.00	9.17
	2020	Non lockdown months	276	92.00	9.64
		Lockdown months	380	126.67	9.50
Niepolomice	2019	Non lockdown months	43	14.33	4.51
		Lockdown months	54	18.00	2.65
	2020	Non lockdown months	47	15.67	3.21
		Lockdown months	35	11.67	4.51
Wieliczka	2019	Non lockdown months	73	24.33	6.35
		Lockdown months	86	28.67	7.51
	2020	Non lockdown months	67	22.33	4.16
		Lockdown months	68	22.67	9.61

SD-Standard deviation.

### AVC patterns in urban versus suburban areas

**Figure 1:**
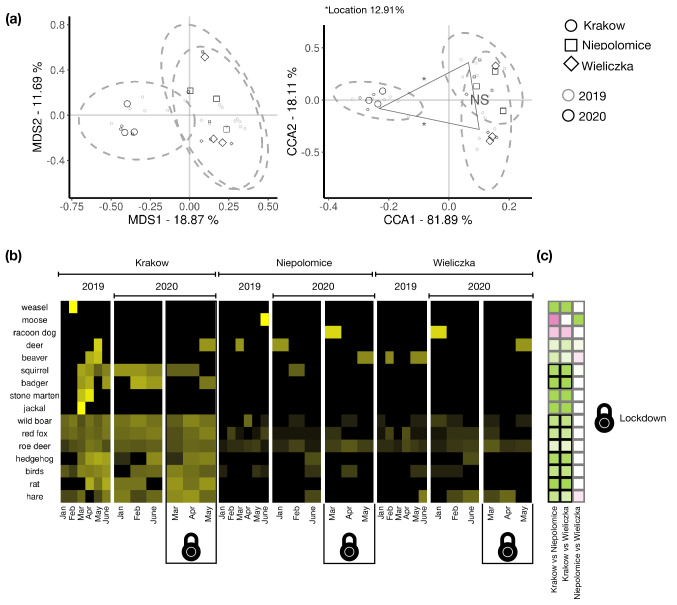
Effect of urbanisation and lockdown on AVC (**a**) Unconstrained ordination shows Multidimensional scaling analysis (MDS) plot (left) displaying the variance explained in component 1 and 2. Constrained ordination (right) showed the variance by location as the constraining factor and year as random factor. The shapes represent the three study locations. The black shapes represent 2020 and grey shapes represent 2019. NS = Not significant; * = *p* ≤ 0.05 represents significance between locations determined by pairwise PERMANOVA. The ellipses represent 95% of the confidence interval; (**b**) The heatmap displaying the percentage of AVC (intensity of colour black to yellow) for the animals reported (y-axis) over a period of month (x-axis) across year for specific location. The lockdown month is facetted out in the year 2020 for the corresponding locations. (**c**) The pairwise comparative statistics performed on location as fixed factors.

**Figure 2:**
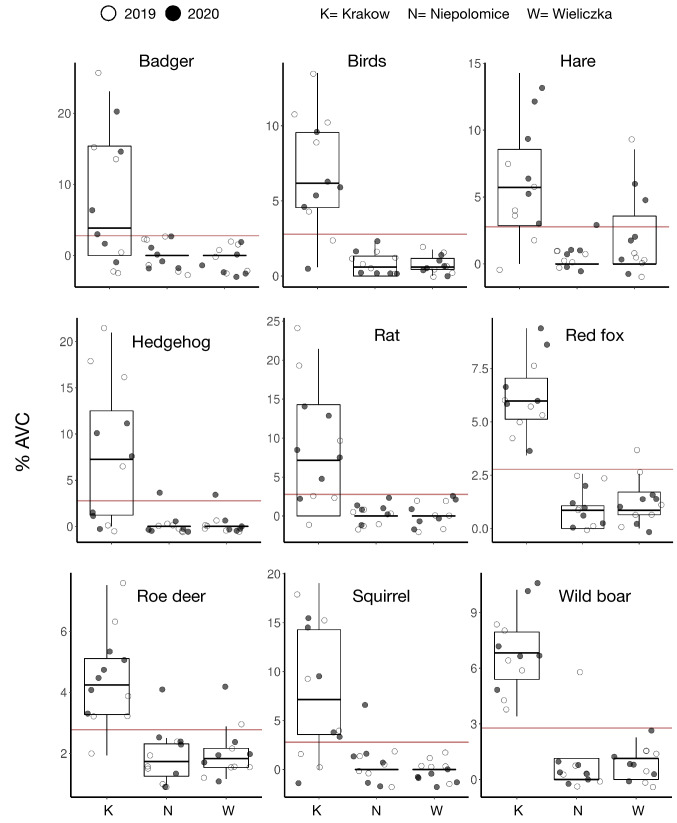
Distribution of AVC (y-axis) of the species with significant difference between the urban and suburban locations over the years (shape fill). The boxes represent the 25th, 50th (median) and 75th percentiles of the data; the whiskers represent the lowest (or highest) datum within 1.5 × interquartile range from the 25th (or 75th) percentile. The horizontal red line indicates the overall mean % of AVC of the specific animal.

To measure variation in AVC across the urban-suburban gradient, Multidimensional Scaling (MDS) was performed with the Jaccard index. The overall variation in AVC was distinct between urban and suburban communes (Fig. 1a). By Canonical Correlation Analysis (CCA), we found that the location factor explained 12.91% of the variance in the dataset (*n* = 36), separating the urban commune of Krakow from the suburban communes of Niepolomice and Wieliczka. The pairwise PERMANOVA found a significant difference (*p* ≤ *0.05*) of variation in AVC between urban and suburban communes, but not significant between suburban communes (Fig. 1a). We found an overall decrease in AVC between 2019 and 2020 in suburban communes, but the AVC rates were similar in Krakow between 2019 and 2020 (Fig. 1b). AVC for red squirrel and European badger (*Meles meles*), wild boar, red fox, roe deer, hedgehog, birds, rats (*Rattus* spp.) and brown hare, were significantly higher in Krakow than in Niepolomice and Wieliczka (Figs. 1c, 2) based on pairwise comparative analysis of AVC among locations.

**Figure 3:**
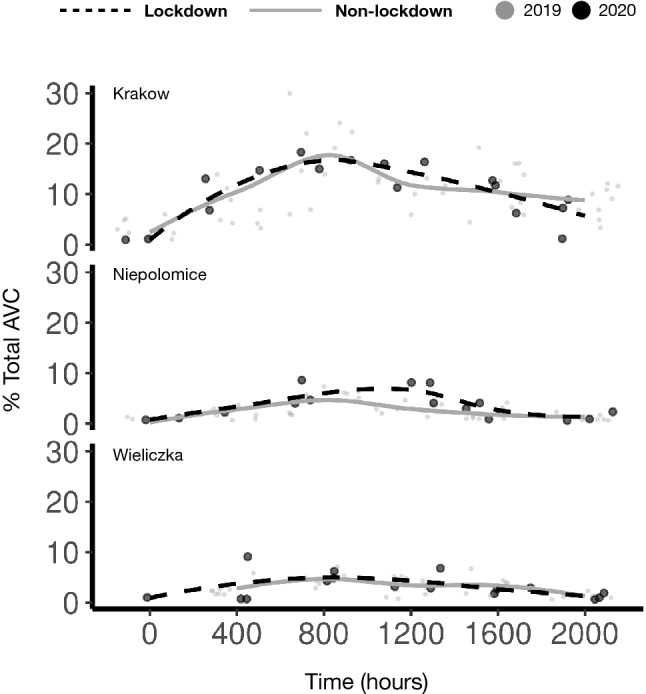
AVC during the lockdown. The LOESS fitted line plot represents the trend of percentage of total AVC (y-axis) across the location along the time (x-axis).

Interestingly, we found a trend in the distribution of total AVC along the time range in different locations. By LOESS fitted line plot (Fig. 3) we found that total AVC were generally higher around 08:00 h and spread until 20:00 h.

### Traffic in Krakow and its impact on AVC

**Figure 4:**
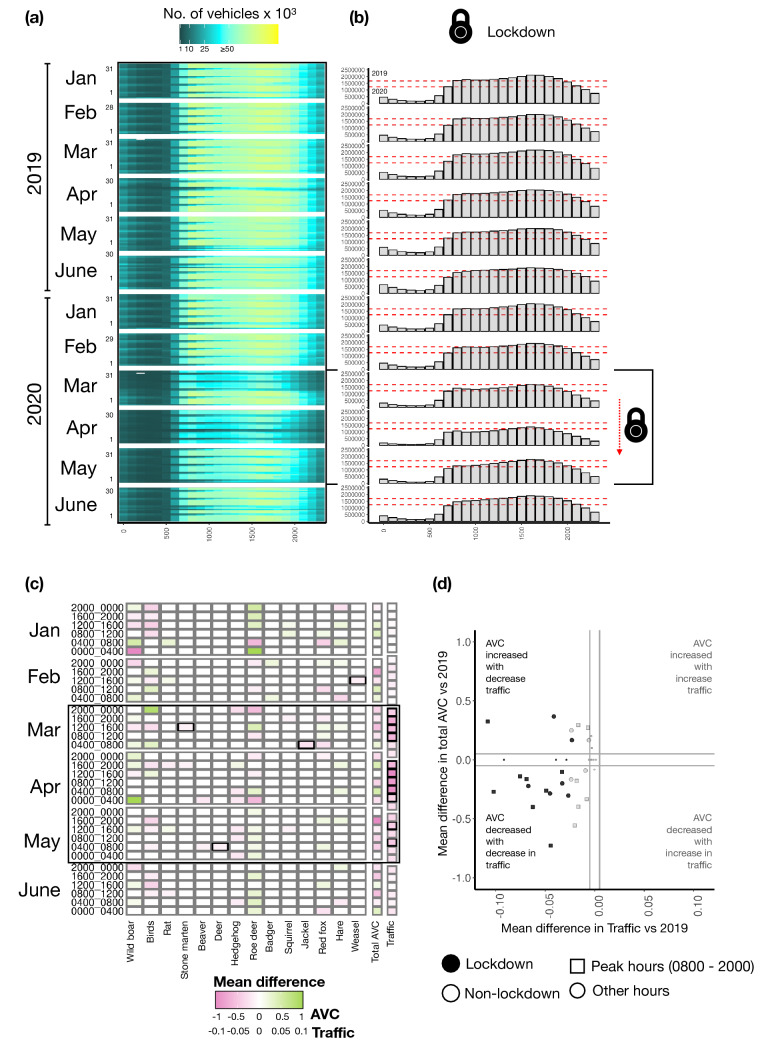
Traffic in Krakow during the lockdown and its impact on AVC (**a**) Heatmap showing the number of vehicles (colour intensity: black, cyan and yellow) in 2019 and 2020 in Krakow from January to June. The x-axis represents hours, and the y-axis represents days; (**b**) Bar plot indicating the overall distribution of the number of vehicles across time for 2019 and 2020 from January to June. The lockdown months are marked by boxes. The red dashed lines indicate the overall median of number of vehicles of 2019 (top) and 2020 (bottom). (**c**) Heatmap using GAM displaying mean difference of AVC (for individual and total animals) and traffic between the 2020 and 2019 in Krakow from January to June within 24 h time period. The lockdown months are facetted; (**d**) Scatterplot showing the mean difference between 2020 and 2019 for AVC (y-axis) and traffic volume (x-axis) by Spearman rank correlation. The x´ and y´ axes represents the |mean difference|≤ 0.01 and ≤ 0.1 respectively (marked in grey lines).

The mean (± SD) number of vehicles per hour between January and June in 2019 was 24,116 (± 11,827.78), whilst in the same period in 2020 it was 20,891 (± 5839.99). The mean number of traffic volume during the lockdown months (March–May) in 2020 was 30,280, while for the same months in 2019, the traffic volume was 43,555. Traffic volume in Krakow decreased significantly during lockdown months (Fig. 4a, b) with April had the highest mean difference in traffic volume (Fig. 4c) between 2019 and 2020. However, despite significantly lower traffic, the total AVC in Krakow neither decrease significantly overall nor during the lockdown (Fig. 4c). The Spearman’s correlation analysis (r_s_ = 0.265; *p* = 0.06) between the mean difference in traffic volume and the mean difference in AVC comparing the two years found a weak relationship (Fig. 4d).

### Spatial patterns change of AVC

**Figure 5:**
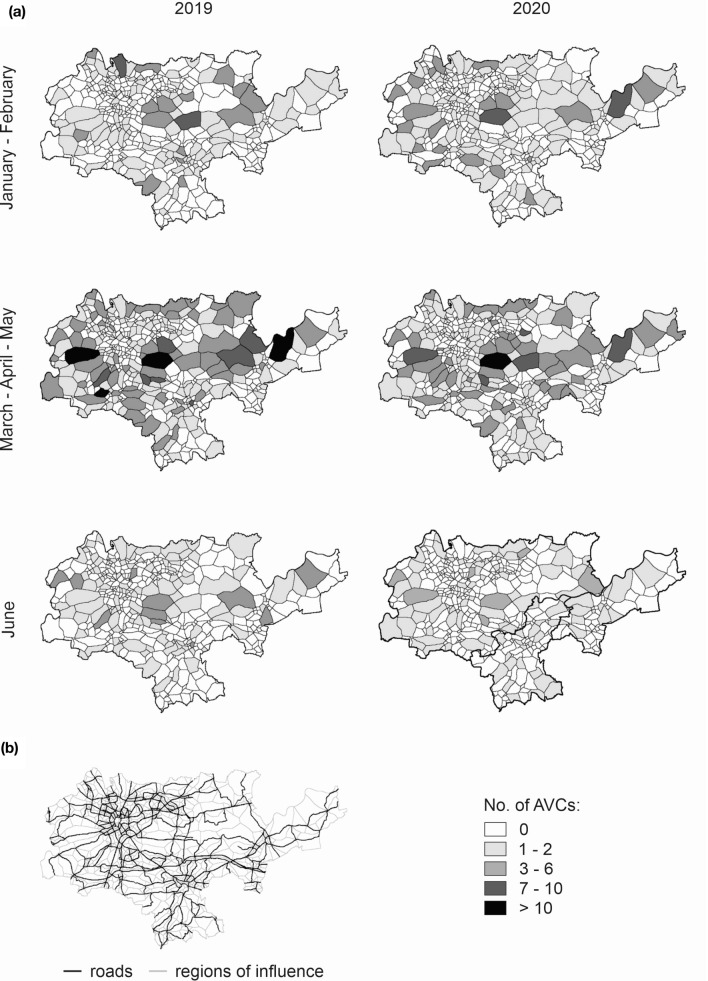
Spatial pattern change of AVC (**a**) Number of AVC incidents reported in regions of influence defined by the network of main streets. (**b**) The network of main streets in the studied communes of Krakow, Wieliczka and Niepolomice.

The spatial pattern of AVC analysed independently for pre-lockdown months (January–February), lockdown months (March–May), and for June differed substantially. The pre-lockdown months in 2020 had a higher number of AVC incidents located in the proximity of forest habitats, in the western and eastern part of the study area in 2020 in comparison to 2019 (Fig. 5). During March–May, AVC were broadly similar between 2019 and 2020 throughout the study area with very minor exceptions in suburban areas in the south. AVC changes in June, by contrast, showed an increase in incidents in 2020 as compared to 2019 in the southern and northern part of the study area, while the east of the study area had a slight decrease in AVC incidents (Fig. 5).

## Discussion

In this study, the urban commune of Krakow had the highest frequency of AVC regardless of the study period (lockdown vs non lockdown). In the suburban communes of Niepolomice and Wieliczka communes, AVC incidents were generally less frequent than in Krakow and were significantly lower during the lockdown period. Our results found that many animal species (i.e., wild boar, red fox, birds, roe deer, hedgehog, brown hare, red squirrel, European badger and rat) were involved in collisions more in the urban study area in comparison to the suburban areas even during lockdown. High rates of AVC may be attributed to several animal-related factors such as behavioural including changes in daily and seasonal activity associated with breeding, foraging or dispersal patterns and also habitat preferences with preferred road-crossing routes^27,28^. Specific foraging behaviour by various species is commonly associated with higher mortality rate on roads as by regularly feeding on roadsides animals are at a greater risk of collisions with vehicles. Most European wild ungulates are forest-dwelling species and are often known for being attracted to roadsides and regularly using road verges searching for specific minerals and plant material^28,29^. Similarly, some predator species frequently forage on roadkill^27,30^ and predate upon prey using road verges as refuges^31^.

Consistent with other studies, we found that AVCs were more pronounced during the early morning hours in Krakow^31–33^, and this trend retained during lockdown. Temporal activity rhythms of species could have a strong influence on the probability of AVC. The majority of mammalian species are crepuscular or nocturnal and are at higher risk of vehicle collision especially from dusk till midnight, and at dawn^34,35^. The most common conflictual wild animals in our study area are wild boar, roe deer and medium sized carnivores such as red fox^36^. On the other hand, roe deer did not show any significant difference in AVC in the three study sites between lockdown and non-lockdown over the 24 h period. In suburban areas, wild boar and red fox were involved in AVC for a shorter time span during lockdown compared to non-lockdown period. This is probably due to the behavioural plasticity in activity patterns that red foxes^37^ and wild boars^38^ exhibit in their native range, which allows both species to adapt to environmental changes. On the contrary, road crossings by roe deer are mainly driven by their behavioural patterns^39,40^ rather than directly by the volume of traffic on a road^41^.

Finally, we found no significant differences in total AVC between lockdown and non-lockdown periods in Krakow, even though there was a significant reduction in traffic volume during lockdown months. Krakow city can be subject to increased animal activity when human impacts are reduced, due to several migration corridors such as the Vistula River that is of regional and European importance, as well as favourable land cover (i.e., western wedge of greenery)^42^. Similar results were found in other studies^22,43^ where AVC did not decrease in all studied regions during COVID-19 related travel restrictions. The relationship between traffic volume and the number of AVC has led to divergent conclusions being presented in studies published thus far. Some studies have found a positive relationship between traffic volume and AVC^44,45^, indicating high road kills during high traffic volumes, while few studies did not find any strong effects^46–48^. Thus, there may not always be a linear relationship between traffic volume and AVC^49^. Our study did not find a strong correlation between traffic volume and AVC in Krakow in 2020 compared to 2019. We therefore suggest that two possibilities for this result. Firstly, animals within the study area responded to lockdown by continuing their activity and movement within urban environments, which resulted in relatively comparable levels of AVC despite the decrease in traffic volume. This explanation is consistent with accounts of various wildlife species making use of human spaces during the pandemic^9^. As such, the decrease in road traffic during the pandemic might have caused certain species of wildlife to tolerate the risks associated with roads to access the benefits of roads and roadsides^43^. On the other hand, the lack of a strong correlation between traffic volume and AVC rates could also be attributed to human driving behaviour rather than animal behaviour alone^43^. In a regional study in Poland^26^, a decrease in drive time was observed across the high-speed roads during the pandemic, which are normally busy during peak hours. Consequently, reduction in traffic volume and empty roads encouraged high speeding during the pandemic^50^. Seiler and Helldin^51^ have argued that low traffic volume coupled with greater vehicular speed can lead to higher animal mortality rates, even during lockdown^43^. This is due to the longer time interval between subsequent rapidly approaching vehicles, which stimulates an animal to attempt road crossings, thereby increasing the likelihood of collision^52^. Therefore, intermediate traffic volumes may result in higher rates of collisions than large traffic volumes because animals may be more willing to attempt to cross roads and highways with moderate than high traffic volume^49^. Furthermore, increased level of stress^53^ or higher alcohol consumption during the pandemic in Poland ^54^ could impair driving thereby affecting collision risks^55^. Thus, a greater understanding of human driving behaviour would also help explain our findings regarding changes in traffic patterns during the pandemic in the urban area.

## Conclusion

In this study we found that a high frequency of AVC existed in our urban study area, even during the lockdown, and that reduced traffic volumes in the city did not correspond to reductions in AVC. Considering these findings within the context of reduced human activity during COVID-19 restrictions, our results suggest that local lockdown measures have had a limited impact on AVC levels within this urban study area. Given the high levels of AVC that were found in this study, the importance of collecting high quality multi-species data, having reliable reporting systems^49^ including the use of AVC hotspot mapping, reduced speed limits, speed bumps and warning road signage in key areas to reduce AVC as a source of human wildlife conflict^56^.

The term ‘anthropause’ was suggested by Rutz et al.^9^ as a label for Covid-19 lockdown, and researchers immediately noted this as a unique opportunity to study human impacts on the biosphere and the Earth’s physical systems^57^. However, the anthropause is also a symbolic and cultural event that might affect how people and governments perceive and act on environmental challenges once the crisis phase of the pandemic has passed^58^. In our study, within the temporal and spatial scales investigated, we suggest that anthropause has impacted urban wildlife populations through a behavioural and ecological ‘release’ mechanism that has increased species’ utilisation of urban habitats, but this release has had limited population effects as mortality, as measured by AVC, was independent of whether the anthropause had occurred. Therefore, there was likely little impacts on biodiversity in relation to AVC within our study area.

## Methods

### Study area

**Figure 6:**
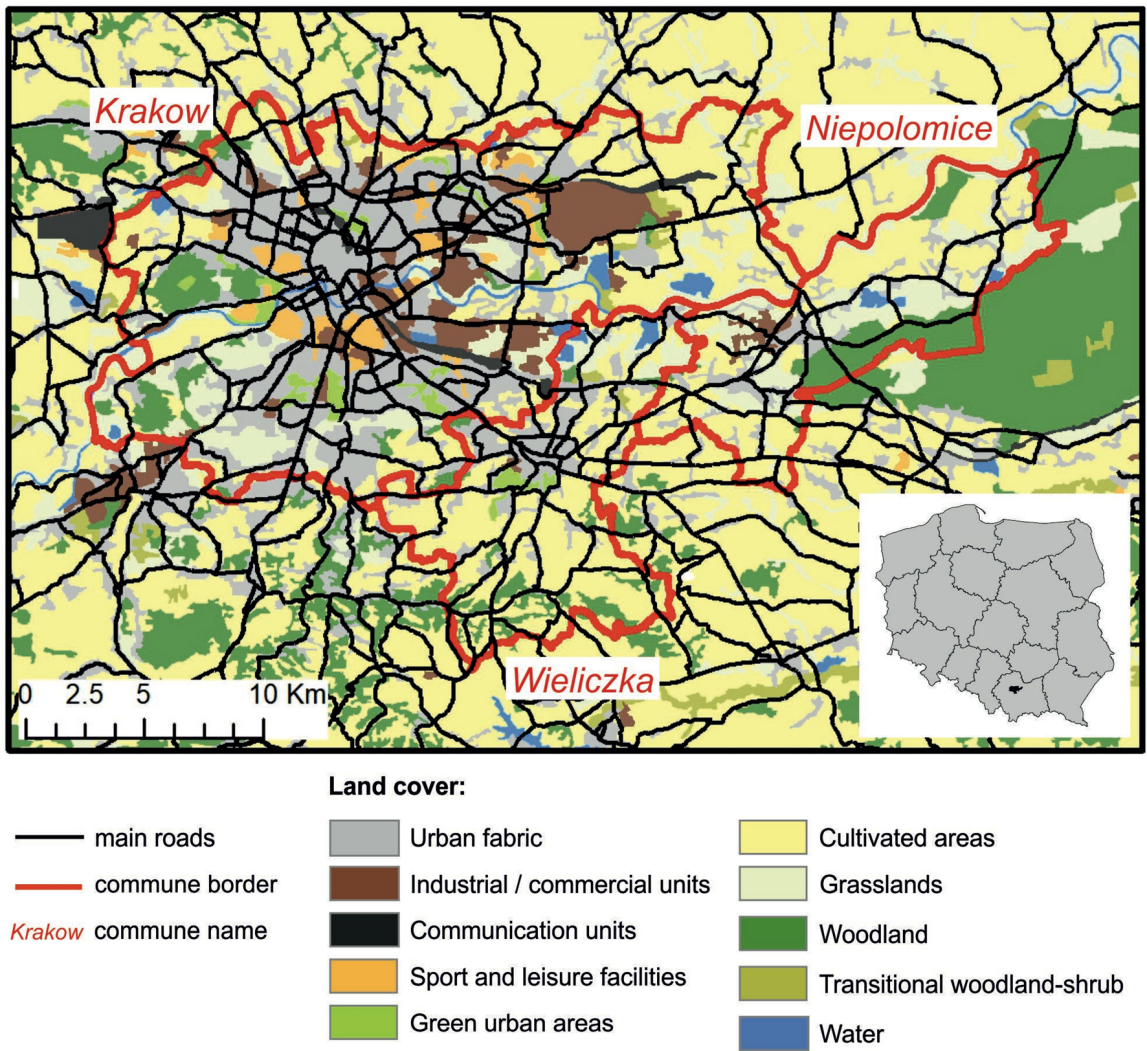
Land cover map of the study area. Land cover information was derived from the CORINE Land Cover 2018 (https://land.copernicus.eu/pan-european/corine-land-cover), and road layer shows all the primary, secondary and tertiary roads from the OpenStreetMap. The figure in the bottom right corner shows location of the study area in Poland.

The study was conducted in southern Poland within the Krakow metropolitan area that comprises three communes of Krakow, Wieliczka, and Niepolomice, representing various levels of urbanisation (Fig. 6, Table 2). The urban commune of Krakow has an area of 327 km^2^ and a human population of 779,115. It is an important transportation hub for major national and international roads, and is bisected by the Vistula River, a natural migration corridor for many wildlife species^59^. In terms of Krakow’s landcover, built-up and urbanised areas constitute over 45% of the city, with 44% of the city used for agricultural purposes including crops, orchards, meadows and pastures.

**Table 2:** Main descriptors of the study areas.

Commune	Level of urbanisation	Total area (km^2^)	Total population	Population density (person/km^2^)	Agricultural land (%)	Forested land (%)	Built-up area (%)
Krakow	Urban	326.9	779,115	2383	43.8	1.7	46.9
Wieliczka	Suburban	99.7	60,781	610	73	7.8	19
Niepolomice	Suburban	96.3	29,141	303	67	15	17

Wieliczka and Niepolomice are mainly agricultural and forested suburban communes neighbouring Krakow (Fig. 6), each covering an area of approximately 100 km^2^, with a human population of 60,781 and 29,141 citizens, respectively. In terms of land cover, built-up and urbanised areas constitute approximately 20% and agricultural land constitutes almost 70% in both suburban communes (Table 2).

Wild animals are common in the study area. In urban parks and in forest patches on the outskirts of the Krakow city, red fox, roe deer and wild boar are found. In Wieliczka, common animals include red deer (*Cervus elaphus*), roe deer, red fox and European badger. Niepolomice commune, on the other hand, encompasses the large Niepolomice forest, a special protection area and Natura 2000 site, inhabited by large wild ungulates including moose (*Alces alces*), red deer, roe deer and wild boar. For this reason, we primarily focused on the effect of lockdown on AVC in wild mammals, AVC for birds were grouped together. Other species, smaller species than birds were not detected among the roadkill.

### Data collection and processing

AVC data was collected daily between January and June of 2019 and 2020 in the study area only by KABAN Company. KABAN Co. were contractually obligated to manage each AVC incident (from birds to large mammals) reported by municipal institutions, such as the Police, City Guard Service, District Centre of Crisis Management and the Krakow Animal Shelter. All these institutions had emergency telephone number that operated 24 h, 7 days a week. KABAN Co., run by Maciej Lesiak who is one of the co-authors of this study, are a professional and highly regulated company, whose staff have considerable scientific expertise in working with wildlife, in particular AVCs. In the study area, AVC incidents must legally be reported immediately, especially in the densely inhabited areas. KABAN Co. officers received phone calls from these institutions about AVC and verified each reported incident by visiting the locations followed by undertaking on-site appropriate procedure e.g., translocating the wounded animal to animal shelter, carcass removal. The reporting time between an AVC incident and the phone calls to KABAN Co was approximately 30 min (Maciej Lesiak personal information). The details of each AVC including the date and location of the incident, the animal species (except for bird species) and their numbers, incident characteristics and the reporting time were recorded in a database (Table S1). Occasionally, multiple records of the same species were reported, for example, if there was more than one individual of the same species being hit on the same day and place. Records of domestic animals were removed from the database. The involvement of KABAN in this study enabled high quality and reliable data collection on AVC, under regulation and legal contractual obligation. Thus, consistent and reliable data on AVCs were available for this investigation, and while we cannot rule out sampling errors or biases per se*,* this study has provided valuable data on the scale and number of species involved in AVCs in this area, and factors underlying variations in AVC that can provide the basis for mitigation and appropriate management.

In original data received from KABAN, the exact point locations were available only for 48% of the AVCs. However, for many entries additional, qualitative location descriptors were available, allowing as to manually assign an accurate location. Due to this procedure, the percentage of AVCs with accurate location increased to 56%. The remaining AVCs were geolocated to street segments, based on street names, which were provided in all reports. Street network data were obtained from the OpenStreetMap (accessed on 29.11.2020), with main streets defined as those of primary, secondary and tertiary road type based on OpenStreetMap typology. The study area was further divided the study area into smaller regions (*n* = 533), where each region contained a main street with smaller streets connected to it and their neighbouring areas (defined using closest Euclidean proximity rule). To be able to investigate changes in spatial pattern of AVCs, the AVC data was aggregated within these regions with regard to year (2019 and 2020) and month (January–February, March–May, and June). June was analysed separately to detect any changes in AVC after strict lockdown was lifted.

Traffic volume data (i.e., number of vehicles per hour) were available only from Krakow and were obtained from the Department of City Traffic through the light detection system installed at major roads for the city of Krakow. The detection system counted the number of vehicles crossing the road on 19 major roads connecting the entire city for the study period on an hourly basis (Table S2).

### Statistical analysis

**Figure 7:**
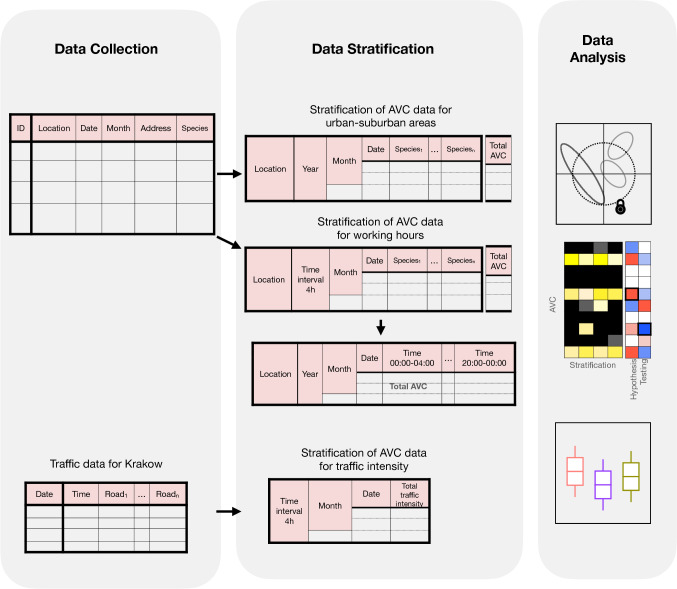
Workflow and data stratification. General workflow for analysing the two research questions.

AVC data relating to the composition of road killed species were analysed using unconstrained (Multidimensional Scaling or, MDS) and constrained (Canonical Correlation Analysis or CCA) ordination methods. Ordination methods are used for multivariate data and can determine differences between samples in a graphical manner. Unconstrained ordination is useful for viewing overall variation in the data (i.e., to represent, the pairwise dissimilarity between objects), whereas constrained ordinations reveal variation of a fixed factor(s) by minimising the effect size of the random factors^60^. All data analyses were performed in the R environment^61^, using tidyverse^62^, Vegan^63^ and RVAideMemoire^64^ packages. All packages and dependencies were encapsulated in an anaconda environment at https://github.com/SAYANTANI26/ProjectAVC/. The detailed data stratification and workflow is available in Fig. 7.

### AVC patterns in urban versus suburban areas

To assess the impact of lockdown months associated with COVID-19 on AVC, the AVC data were stratified by location (understood as commune, i.e., Krakow, Wieliczka and Niepolomice), month (January, February, March, April, May, and June), and year (2019 and 2020) (see for data stratification Fig. 7). The stratified data was normalised and scaled to 1 for all further analysis. The MDS analyses were performed by computing the dissimilarity in AVC using Jaccard index^65^. The dataset was further estimated by CCA using location as a constrained variable and conditioned by year. Statistical significance (*p* ≤ 0.05) of locations were determined by performing PERMANOVA and the variation within location by pairwise PERMANOVA (over 1000 permutations). The *p-*value for the pairwise analysis was adjusted using the Benjamini–Hochberg (BH) procedure. Next, we represented the AVC patterns for each species (mammals and birds) using heat maps for visual inspection. Heat maps enabled visualisation of the intensity (high or low) of AVC for each species in the three locations. Finally, by generalised additive model (GAM) using Poisson distribution (Eq. 1), we analysed the mean difference of AVC between locations and lockdown (fixed factors) (Eq. 2). The statistical significance was computed by conducting Tukey’s Honest Significance Difference (HSD) test and the *p*-value was adjusted using the BH procedure. Animal species that had a mean difference with false discovery rate (FDR) ≤ 0.05 were considered to be significant and the percentage of AVC for those animals was represented in boxplots.

1 $${\text{Poisson}}\left( {f\left( x \right)} \right) = y_{{\text{AVC of animal}}}$$

2 $$f\left( x \right) = x_{{{\text{location}}}} + x_{{{\text{lockdown}}}} + {\text{random}}\left( {x_{{{\text{year}}}} \times x_{{{\text{month}}}} } \right)$$

3 $$f\left( x \right) = x_{{{\text{year}}}} \times x_{{{\text{month}}}} \times x_{{\text{time range}}}$$

To assess the AVC on an hourly scale, data were grouped by summing the AVC reports for each day and each month within the 24-h (h) time period of the corresponding location (commune). The time period was divided into six intervals of 4 h time periods (00:00–04:00 h, 04:01–08:00 h, 08:01–12:00 h, 12:01–16:00 h, 16:01–20:00 h and 20:01–00:00 h). Additionally, the total AVC across time was estimated by stratifying month, year and location (see for data stratification Fig. 7). The trend of variation in total AVC along the time range was represented by fitting the total AVC by the locally estimated scatterplot smoothing (LOESS) method.

### Traffic in Krakow and its impact on AVC

Finally, to assess the influence of traffic volume (i.e., number of vehicles per hour) on AVC during the lockdown, the traffic and the AVC dataset (stratified by month and 24 h time period) for Krakow were integrated to analyse the association of vehicle movement and its impact on AVC. Traffic volume data was normalised by dividing the number of vehicles (per hour) by 1 000 000 (in million) to optimise the mean differences within the range of |0–1|. Thus, a difference of − 0.01 would correspond to a decrease of 10 000 vehicles of corresponding months in 2020 in comparison to 2019. We independently compared the datasets for each parameter (animal species, total AVC and traffic volume) within Krakow between the same months of 2019 and 2020 (fixed factor) and lockdown. Using GAM, the effect of lockdown and year for each parameter was analysed and the statistical significance was reported (same as in 3.2.1; Eq. 3). The relationship between traffic volume (converted to per million within 24 h time period) and AVC was determined by conducting Spearman's rank correlation (rho) on the respective mean difference.

## Supplementary Information

Supplementary Information 1.

Supplementary Information 2.

## Data Availability

Datasets of all spectra shown are available in: R scripts used to generate figures are available at: https://github.com/SAYANTANI26/ProjectAVC.
