# Supplementary material for: Animal-vehicle collisions during the COVID-19 lockdown in early 2020 in the Krakow metropolitan region, Poland
Source: Sci Rep. 2022 May 9;12:7572. doi: 10.1038/s41598-022-11526-9 (PMC9082987; doi:10.1038/s41598-022-11526-9)
Supplement: Supplementary file 2 — Supplementary Information 2. [file 41598_2022_11526_MOESM2_ESM.docx]

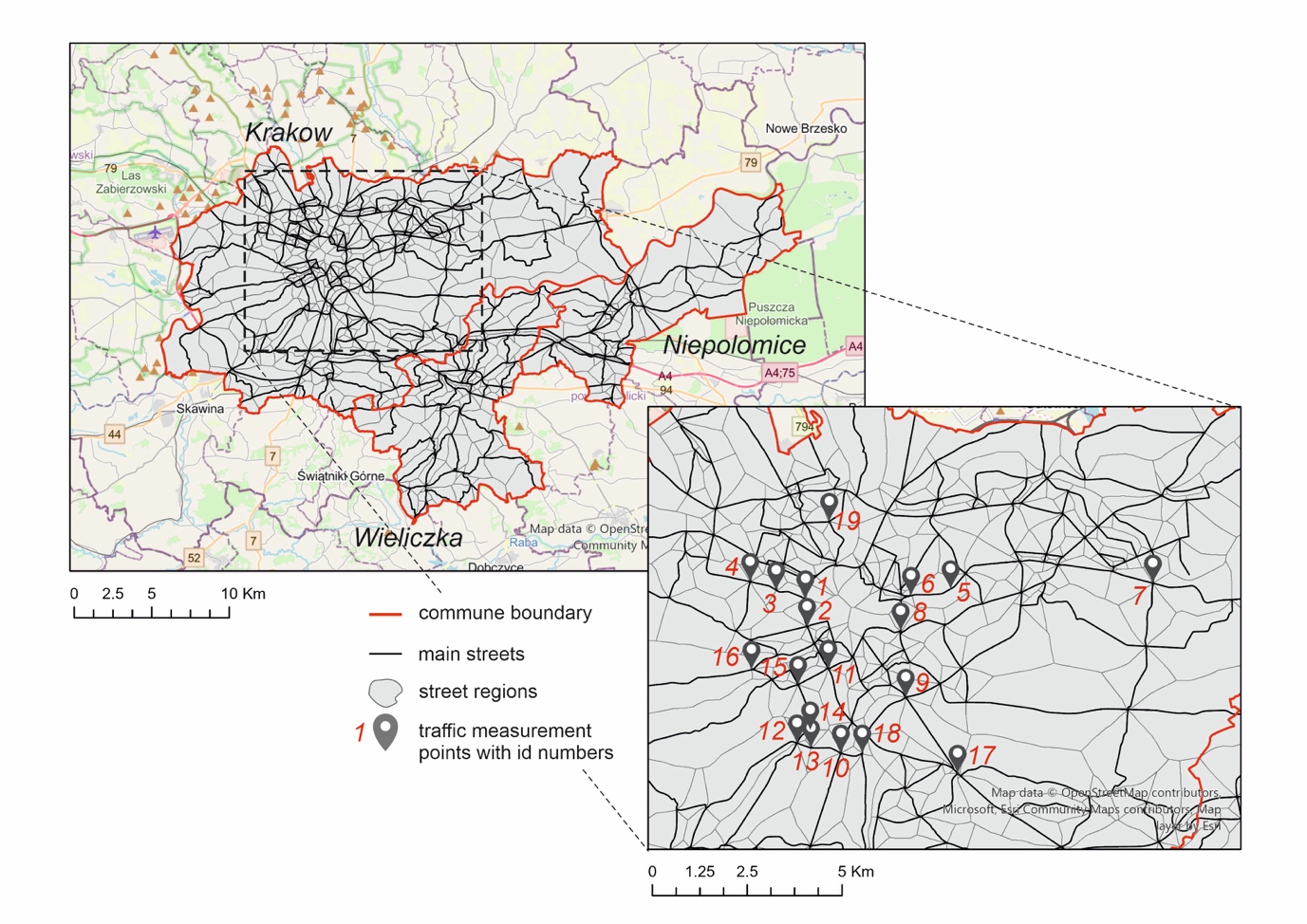


Fig. S1 Traffic volume measurement points along 19 major road crossings in Krakow. Road layer shows all the primary, secondary and tertiary roads from the OpenStreetMap. Administrative boundaries obtained from the National Register of Boundaries in Poland (www.geoportal.gov.pl). Traffic measurement points generated based on data obtained from the Department of City Traffic in Kraków. Data analysed with ArcMap 10.8.1, <https://desktop.arcgis.com/en/arcmap/latest/get-started/main/get-started-with-arcmap.htm>.
